# Recruiting women with ductal carcinoma in situ to a randomised controlled trial: lessons from the LORIS study

**DOI:** 10.1186/s13063-023-07703-4

**Published:** 2023-10-14

**Authors:** Sally Wheelwright, Lucy Matthews, Valerie Jenkins, Shirley May, Daniel Rea, Pat Fairbrother, Claire Gaunt, Jennie Young, Sarah Pirrie, Matthew G. Wallis, Lesley Fallowfield, on behalf of the LORIS Trial Management Group, John M. S. Bartlett, Lucinda Billingham, Sarah Bowden, Samantha Brace-McDonnell, Cassandra Brookes, Henry Cain, David Dodwell, Andrew Evans, Patricia Fairbrother, Douglas Ferguson, Adele Francis, Andrew Hanby, Fiona Hoar, Simon Holt, Cliona Kirwan, Stuart McIntosh, Sarah E. Pinder, Malcolm Reed, Tracy Roberts, Jennifer Rusby, Nisha Sharma, Pauline Sibley, Jeremy Thomas, Maggie Wilcox

**Affiliations:** 1 Sussex Health Outcomes Research & Education in Cancer (SHORE-C), Brighton & Sussex Medical School, University of Sussex, Falmer, Brighton, BN1 9RX UK; 2 Cancer Research UK Clinical Trials Unit, Institute of Cancer and Genomic Sciences, University of Birmingham, Birmingham, B15 2TT UK; 3 Independent Cancer Patients’ Voice, London, UK; 4 Cambridge Breast Unit and NIHR Cambridge Biomedical Research Centre, Addenbrooke’s Hospital, Cambridge, CB2 2QQ UK

**Keywords:** DCIS, LORIS, Patient preference, Patient interviews, Trials, Randomisation

## Abstract

**Background:**

The LOw RISk DCIS (LORIS) study was set up to compare conventional surgical treatment with active monitoring in women with ductal carcinoma in situ (DCIS). Recruitment to trials with a surveillance arm is known to be challenging, so strategies to maximise patient recruitment, aimed at both patients and recruiting centres, were implemented.

**Methods:**

Women aged ≥ 46 years with a histologically confirmed diagnosis of non-high-grade DCIS were eligible for 1:1 randomisation to either surgery or active monitoring. Prior to randomisation, all eligible women were invited to complete: (1) the Clinical Trials Questionnaire (CTQ) examining reasons for or against participation, and (2) interviews exploring in depth opinions about the study information sheets and film. Women agreeing to randomisation completed validated questionnaires assessing health status, physical and mental health, and anxiety levels. Hospital site staff were invited to communication workshops and refresher site initiation visits to support recruitment. Their perspectives on LORIS recruitment were collected via surveys and interviews.

**Results:**

Eighty percent (181/227) of eligible women agreed to be randomised. Over 40% of participants had high anxiety levels at baseline. On the CTQ, the most frequent most important reasons for accepting randomisation were altruism and belief that the trial offered the best treatment, whilst worries about randomisation and the influences of others were the most frequent most important reasons for declining. Most women found the study information provided clear and useful. Communication workshops for site staff improved knowledge and confidence but only about half said they themselves would join LORIS if eligible. The most common recruitment barriers identified by staff were low numbers of eligible patients and patient preference.

**Conclusions:**

Recruitment to LORIS was challenging despite strategies aimed at both patients and site staff. Ensuring that recruiting staff support the study could improve recruitment in similar future trials.

**Trial registration:**

ISRCTN27544579, prospectively registered on 22 May 2014

**Supplementary Information:**

The online version contains supplementary material available at 10.1186/s13063-023-07703-4.

## Background

Ductal carcinoma in situ (DCIS) is a condition in which neoplastic breast cells are confined to the lining of breast ducts. It is classified into three histological grades—high, intermediate, and low—according to cyto-nuclear feature [1]. Whilst high-grade DCIS (typically grade 2 or 3) may progress to invasive cancer, there is greater uncertainty surrounding the behaviour of low- and low-intermediate-grade DCIS [1]. The occurrence of DCIS increased dramatically following the introduction of breast cancer screening programmes in the 1980s. In the USA, for example, DCIS incidence rose from 1.87 per 100,000 in 1973–1975 to 32.5 in 2004 [2]. Approximately 20% of screen detected breast cancers are DCIS [3].

In 2012, an independent panel reviewed the evidence on benefits and harms of screening in the context of the UK breast screening programmes. The resulting Marmot report concluded that whilst breast screening may extend lives, it also detects cancers which would not come to clinical attention during a woman’s life; such overdiagnosis has a negative impact on quality of life [4]. The report recommended research to improve prognosis prediction and treatment of DCIS. At present, although calls continue for low-grade DCIS to be downgraded from Stage 0 cancer to pre-cancer, worthy of being watched closely but not automatically treated [5–7], surgery remains the standard treatment for all grades of DCIS, often followed by radiotherapy and sometimes with endocrine treatment.

The LOw RISk DCIS (LORIS) study was set up to examine if *not* treating DCIS is safe [8]. Three other randomised controlled trials (RCTs)— Comparison of Operative versus Monitoring and Endocrine Therapy Trial |(COMET) in America [9], LOw Risk DCIS (LORD) Study in the Netherlands [10], and low-risk DCIS with endocrine therapy alone-tamoxifen (LORETTA) in Japan [11]—have been set up subsequently. Each of these trials set out to randomise DCIS patients between conventional treatment and active monitoring with annual mammography. Recruitment to trials which seem to be comparing ‘something’ with ‘nothing’ is known to be challenging [12]. Consequently, LORIS utilised a number of strategies to maximise recruitment by supporting both patients and recruiting sites. These methods are described here together with the reasons women gave for their decisions on whether to participate.

## Methods

The LORIS study is a multi-centre, phase III RCT of surgery versus active monitoring with annual mammography in patients with low-risk ductal carcinoma in situ (DCIS) [8]. LORIS was open to recruitment from July 2014 to March 2020, with a 2-year internal feasibility phase, and included 49 sites. The primary aim of LORIS was to assess whether active monitoring is non-inferior to surgery, defined by ipsilateral invasive breast cancer-free survival. The aim of this paper is to describe the recruitment strategies adopted in LORIS and to identify why women did and did not agree to participate.

### Inclusion and exclusion criteria

Women aged ≥ 46 years with a histologically confirmed diagnosis of non-high-grade DCIS (unilateral or bilateral) in the last 90 days who were fit and willing to undergo surgery and able to provide written informed consent were eligible. Exclusion criteria included a previous diagnosis of ipsilateral DCIS or invasive breast cancer, a mass lesion not proven on biopsy to be a specific benign lesion, unequivocal comedo necrosis observed, recent onset ipsilateral blood-stained nipple discharge without benign explanation or being in a high-risk group for developing breast cancer.

### Registration and randomisation pathway

**Fig. 1:**
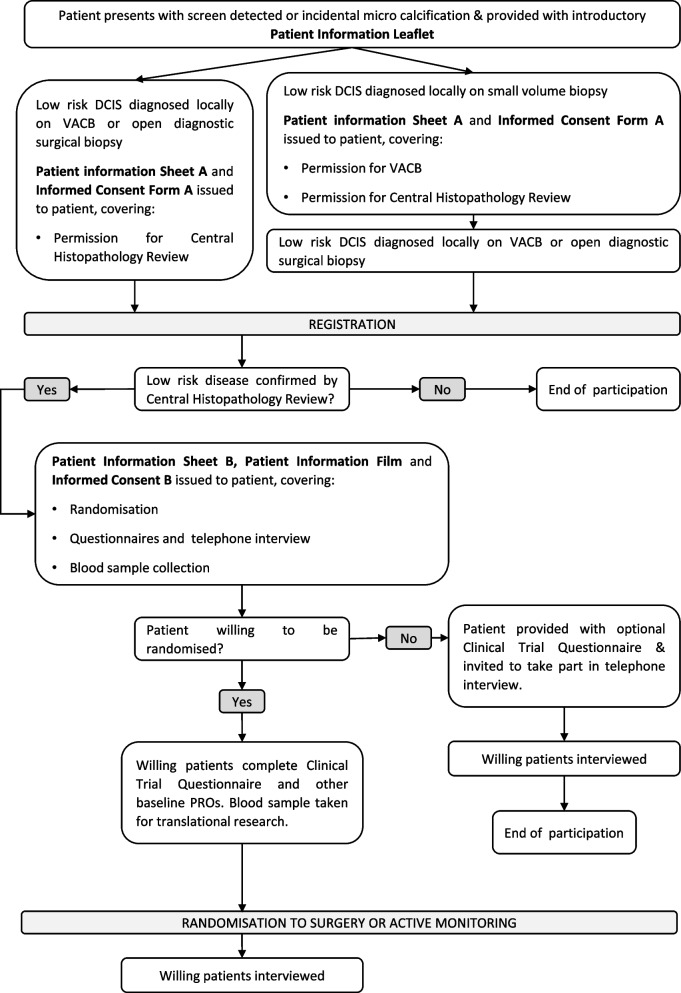
Registration and randomisation pathway

Enrolment to LORIS was a two-step process; registration followed by randomisation (see Fig. 1). Women who provided informed consent were registered if low-risk DCIS was diagnosed locally following vacuum-assisted core biopsy (VACB) or open diagnostic surgical biopsy. These procedures were standard care at some sites, whereas at other centres, small volume biopsy was the standard and women provided additional consent for VACB. All diagnostic histology slides were sent for digital scanning and central histopathology review by the trial’s pathologists.

Women were eligible for randomisation into LORIS if a diagnosis of low-risk DICS was confirmed by central review. Those eligible for LORIS were consented and randomised 1:1 to either surgery, the contemporary standard treatment for DCIS, or active monitoring, i.e. annual mammograms for 10 years. Women who declined randomisation were invited to complete the Clinical Trials Questionnaire (CTQ), a widely used validated measure of reasons why patients agree or decline entry into RCTs [12], and participate in a 15-min telephone interview. Women who agreed to be randomised were asked to complete the following validated measures pre-randomisation Spielberger State Trait Anxiety Inventory (STAI) [13]; EuroQOL 5D-5 L (EQ-5D) [14] and the Short Form Health Survey-36 version 2 (SF-36v2) [15], along with the CTQ, and were invited to participate in a 15-min interview post randomisation.

### Measures

#### Clinical Trial Questionnaire (CTQ)

Reasons for accepting or declining LORIS were collected using the CTQ [12]. Question one establishes whether the patient has agreed to trial entry, followed by 16 possible reasons that may influence participation. Respondents indicate on a Likert scale their agreement with each statement: 0 = strongly agree; 1 = agree to some extent; 2 = unsure; 3 = disagree to some extent; and 4 = strongly disagree, and select the most important reason for their choice. They are also invited to list any other reasons for their decision.

#### Spielberger State Trait Anxiety Inventory (STAI)

The STAI consists of two sets of 20 questions, each scored on a 4-point scale [13]. One set provides a measure of trait anxiety (underlying predisposition to anxiety) whilst the other set assesses state anxiety (current anxiety levels). Higher scores indicate higher anxiety. Various cutoffs for significant levels of anxiety have been proposed; a score of 40 or higher was chosen for this study [16].

#### EuroQol 5D-5 L (EQ-5D)

The EQ-5D is a health status measure, comprising five dimensions (mobility, self-care, usual activity, pain/discomfort, and anxiety/depression) [14]. Each dimension is scored between one and five. A profile of 11111 indicates full health whilst 55555 is the worst possible state. In addition, respondents are asked to mark their self-rated health on a visual analogue scale, ranging from 0 (the worst health you can imagine) to 100 (the best health you can imagine).

#### Short Form Health Survey- 36 version 2 (SF-36v2)

The SF-36v2 is a health status measure covering eight domains: physical functioning, role participation with physical health problems, bodily pain, general health, vitality, social functioning, role participation with emotional health problems and mental health [15]. Response options vary across items. All eight domains contribute to a physical component summary (PCS) measure and a mental component summary (MCS) score. Benchmarking is provided by a 1990 US general population.

### Patient interviews

Semi-structured interviews were conducted with women willing to be randomised and those who were not (see Supplementary Information for interview details). The interview schedule comprised four sections: (1) basic demographics; (2) whether any health care professionals (HCPs) influenced decision to take part or not; (3) trial information received (patient information sheets and film) and thoughts about it; (4) expanding on the most important reason they accepted/declined LORIS study. Interviews were conducted post randomisation. There were some minor changes to interviews over the course of the study, as described in the “Results” section.

### Study information

Written materials and a film were produced to help women’s decision making about participation. Patient and public focus groups were held to inform the content of these materials [17]. All mentions of DCIS used the most preferred description from a survey completed by 54 healthcare professionals attending a British Breast Group meeting [18]. A patient information leaflet (PIL) was provided pre-biopsy to introduce the concept of the study. Before registration, Patient Information Sheet A (PIS-A) provided information about VACB and the central review process. Prior to randomisation, women received Patient Information Sheet B (PIS-B) and a patient information film. The film was 30 min long and available in DVD format, via YouTube and on the LORIS website [19].

The film was divided into chapters for ease of re-viewing particular sections or could be played in full (see Supplementary Information for list of chapters). The trial was presented in a balanced manner, using lay language with simple graphics to demonstrate concepts such as randomisation [20]. Also included was an interview with the Chief Investigator (CI) and a question and answer (Q&A) session, featuring clinicians answering women’s questions about the study. LORIS recruitment sites provided access to the film to women following their own trial discussions.

### Support for sites

At the start of the project, communication workshops were delivered regionally to recruiting sites’ staff (see Supplementary Information for the workshop protocol). The focus of these were informed by previous research [21–23], on how best to describe DCIS and randomisation. Difficult questions and communicating with family members were covered during role play scenarios with simulated patients (actors). Pre- and post-workshop, participants completed questionnaires, probing knowledge about the trial, confidence when discussing clinical trials in general and randomisation specifically (see Supplementary Information for the pre-workshop version of the questionnaire).

A year into the main trial, a funder monitoring visit highlighted recruitment rate concerns so a recovery plan was developed to increase recruitment. Postal surveys were sent to PIs, site leads, and then other members of the recruiting site teams. The surveys explored which specific aspects of the LORIS protocol were most challenging. In parallel to surveys, all PIs and site leads were invited to participate in semi-structured telephone interviews to discuss further recruitment challenges. The information derived from surveys and interviews helped identify those teams likely to benefit from either communication workshops or refresher site initiation visits (SIVs). For example, those raising concerns about discussing randomisation, dealing with patient preferences for different management, or HCPs with negative attitudes towards LORIS, were invited to communication workshops. Refresher SIVs were offered to teams who had experienced organisational changes, or who had diagnostic or pathway logistic challenges. In addition, a LORIS awareness poster and leaflet were created and made available within hospitals, breast screening units, published within the Association of Breast Surgeons bulletins and shared to Linked-In and Twitter.

### Statistical analyses

SPSS Statistics version 26 (IBM SPSS Statistics for Windows, Version 26.0. Armonk, NY: IMB Corp) was used to produce descriptive statistics. Differences between acceptors (women with confirmed low-risk DCIS who were willing to be randomised) and decliners (women with confirmed low-risk DCIS who were not willing to be randomised) in endorsement of the statements on the CTQ were compared using chi-squared test or Fisher’s exact test as applicable. Paired sample *t*-tests were used to compare scores on the pre- and post-communication workshop questionnaires.

## Results

**Fig. 2:**
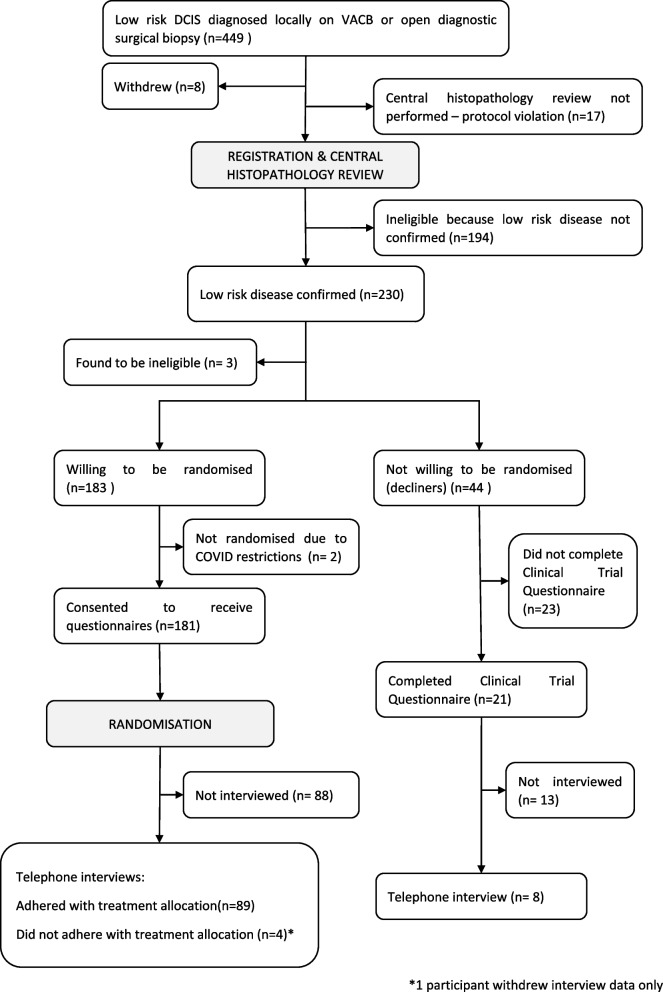
CONSORT flow diagram

The CONSORT flow diagram is shown in Fig. 2. Of the 227 eligible women who had confirmed low-risk DCIS, 183 (81%) accepted randomisation and 44 (19%) declined. Two acceptors could not be randomised due to COVID-related issues, so 181 acceptors were randomised. The CTQ was completed by 175/181 (97%) acceptors and 21/44 (48%) decliners. Telephone interviews were conducted with 101 women, including 92/181 (51%) acceptors, and 8/44(18%) decliners. At the time of interview, four of the women who had been randomised to standard treatment had decided not to go ahead with surgery. One of the four also withdrew her interview data so is not included in that analysis below.

### Participants

The median age of the 181 acceptors was 58 years (range 46–88 years) and most were post-menopausal (117/181, 65%, post-menopausal; 19/181, 10%, peri-menopausal; 34/181, 19% pre-menopausal; 11/181, 6% menopausal status not known). The median age of the 92 acceptors interviewed post randomisation was 60 years (range 48–88 years), 62/92 (67%) had a partner and 55/92 (60%) were employed. The median age of the 8 decliners interviewed post randomisation was 55 years (range 48–70 years), 5/8 (63%) had a partner and 5/8 (63%) were employed.

Table 1 summarises scores on the STAI, EQ-5D and the SF36-v2 for acceptors. For STAI-trait, 53/174 (30%) scored 40 or above, indicating high trait anxiety and for STAI-state, 75/175 (43%) scored 40 or above indicating high state anxiety. On the EQ-5D, 61/176 (35%) had full health (a profile of 11111) and no women were in the worst possible state (55555). On the SF36-v2, 141/174 (81%) had a PCS measure which was the same or better than the general population. For the MCS measure, 122/174 (70%) were the same or better than the general population.

**Table 1:** Baseline measure scores for women who consented to randomisation

**Measure (minimum, maximum possible scores)**	***N***	**Mean**	**Standard deviation**	**Median**	**Range**
STAI Trait (20, 80)	174	35.2	9.9	34.0	20–64
State (20, 80)	175	37.7	11.8	37.0	20–68
EQ-5D VAS (0, 100)	176	83.1	14.6	85.0	20–100
SF36-v2 PCS (0, 100)	174	52.7	8.7	55.7	20.3–68.9
MCS (0, 100)	174	50.2	9.6	52.4	23.2–67.6

### Clinical Trials Questionnaire

Table 2 displays the frequency of agreement (‘strongly agree’ or ‘agree to some extent’) with each reason and the frequency of endorsement as the most important reason, according to whether participants accepted or declined to be randomised. Although significantly more decliners were concerned about randomisation (76%) and wanted the doctor to choose their treatment (71%), nearly half of acceptors were also worried about randomisation and 39% wanted the doctor to choose their treatment.

**Table 2:** Clinical Trials Questionnaire responses

**Reason**	**Frequency of agreement (‘strongly agree’ or ‘agree to some extent’) with each reason for accepting or declining trial participation**				**Most important reason**	
	**Acceptors** ***n* = 175**	**Decliners** ***n* = 21**	***χ***^**2**^	***P* value**	**Acceptors** ***n* = 175**	**Decliners** ***n* = 18**
I thought the trial offered the best treatment available	159 (91%)	6 (29%)	54.6	< 0.001^a^	35 (20%)	1 (6%)
I believed the benefits of treatment would out-weigh any side effects	142 (82%)^b^	1 (5%)	56.6	< 0.001	5 (3%)	0 (0%)
I was satisfied that either treatment in the trial would be suitable for me	141 (81%)	6 (29%)	27.0	< 0.001	7 (4%)	0 (0%)
I was worried that my illness would get worse unless I joined the study	29 (17%)	1 (5%)^b^	1.8	0.3^a^	0 (0%)	1 (6%)
The idea of randomisation worried me	84 (48%)	16 (76%)	6.0	0.02	1 (1%)	3 (17%)
I wanted the doctor to choose my treatment rather than be randomised by computer	69 (39%)	15 (71%)	7.8	0.005	0 (0%)	1 (5%)
The doctor told me what I needed to know about the trial	171 (98%)	19 (91%)	3.3	0.1^a^	0 (0%)	0 (0%)
I trusted the doctor treating me	173 (99%)	19 (95%)^b^	1.8	0.3^a^	7 (4%)	0 (0%)
I was given too much information to read about the trial	29 (17%)	0 (0%)	4.1	0.05^a^	0 (0%)	0 (0%)
I was given enough information to read about the trial	170 (97%)	17 (81%)	11.2	0.009^a^	6 (3%)	3 (17%)
I knew I could leave the trial at any time and still be treated	175 (100%)	18 (86%)	25.4	< 0.001^a^	23 (13%)	2 (11%)
I did not feel able to say no	13 (7%)	1 (5%)	0.2	1.000^a^	0 (0%)	0 (0%)
I wanted to help the doctors research	166 (95%)	11 (55%)^b^	34.0	< 0.001^a^	34 (19%)	1 (6%)
I feel that others with my illness will benefit from results of the trial	174 (99%)	12 (57%)	69.0	< 0.001^a^	55 (31%)	0 (0%)
The doctor wanted me to join the trial	62 (35%)	5 (25%)^b^	0.9	0.4	1 (1%)	1 (6%)
Others (e.g. family or friends) wanted me to join the trial	83 (47%)	1 (5%)	13.9	< 0.001	1 (1%)	5 (28%)

^a^Fisher’s exact test

^b^1 missing response

There were nine statements which significantly more acceptors agreed with, including the belief that the trial offered the best treatment available (91% vs. 29%), and wanting to help both the doctor’s research (95% vs. 55%) and others with the same illness (99% vs. 57%). These three reasons were also most frequently chosen as the most important reason for agreeing to be randomised. For decliners, the most frequently cited key reasons were the influence of others, such as family or friends (28%) and the idea of randomisation being worrying (17%). Most women, regardless of whether they agreed to be randomised, felt they could trust the doctor providing treatment (98%) and that s/he had told them what they needed to know about the trial (97%).

Most participants who listed other reasons for agreeing to be randomised indicated they thought the trial offered the best (safest) way of avoiding surgery. Some noted the benefits of extended monitoring. Whilst many LORIS participants found randomisation worrying, two women noted that they saw this as a benefit as they were undecided about whether they wanted surgery or not. Most of the women who declined randomisation and offered an additional reason, made it clear that they definitely wanted surgery. For one woman, the opposite was true—she did not want surgery.

### Interview results

#### Reasons for accepting or declining randomisation

Interviews took place a median of 22 days after randomisation. During the interview, the women were given the opportunity to elaborate further on the reasons they had given on the CTQ for accepting or declining randomisation. The three most frequent reasons for accepting randomisation were the same for this subset of 89 women as the 175 who completed a CTQ. Anticipating future benefit for others was selected by 19/89 (21%) as the most important factor, e.g. “I’ve got 9 grandchildren and 6 of them are girls. What if same thing happens when they grow up and the only option was the operation? I hope that anything I do helps, not just for my family, but all women.” (P24). Helping with the doctor’s research was also selected by 19/89 (21%), e.g. “Well it’s like anything else, if we don’t have individuals giving their time and biopsy samples, we’ll never find out if these dormant cells will turn into invasive cancer. It will help future generations, I think this is what you need. I think there should be more research in general and more money should be spent on research. My husband was worried that I’d get monitoring, but I was happy to join either part of the trial.” (P102). And for another 19/89 (21%), the most important reason for accepting randomisation was the belief that the trial offered the best treatment e.g. “I thought getting yearly rather than 3 yearly mammograms was a good idea. Getting checked more often was the main reason.” (P48).

The four women who withdrew from their treatment allocation after randomisation had all been allocated to standard treatment but did not want surgery. For example, one woman talked about how surgery did not fit in with her busy lifestyle as a self-employed fitness instructor and that she felt it was currently unnecessary. Nearly all the decliners interviewed (7/8) were sure they wanted surgery, generally for peace of mind or because of family considerations, with just one woman declining randomisation because she did not want surgery.

#### Information provision

For 83/89 (93%) acceptors who were interviewed, the trial had been discussed with more than one HCP, including research nurses (82/89, 92%), surgeons (75/89, 84%), radiologists (11/89, 12%), and an oncologist (1/89, 1%). Most acceptors (83/89, 93%) did not feel that their decision to take part had been influenced by their discussion with an HCP. Similarly, all but one of the decliners had discussed the trial with more than one HCP. Two out of the nine women in this group felt their decision had been influenced at least to some extent by an HCP: one because the surgeon had made it clear that the patient had to be content with both options on offer before entering the trial and one because the surgeon had mentioned surgery before anything else.

All the women who were interviewed remembered receiving and reading at least one of the three written information documents: 89/100 (89%) reported reading the PIL, 91/100 (91%) reported reading PIS-A and 97/100 (97%) reported reading PIS-B. Some women talked about reading everything very carefully, and even going online to find out more about both DCIS and the LORIS study or sharing the information with friends who were HCPs. Others described skimming the leaflets or just reading what they considered to be important. One woman explained how she had been too fearful to read the trial sheets initially but had felt comforted about the whole process after a nurse went through everything verbally. Another individual initially found the information too much but followed a friend’s advice to read each PIS bit by bit.

Further interview questions focussed specifically on PIS-B, which explained randomisation. Most of the women (73/100, 73%) shared this leaflet with someone else, usually family members but also friends and colleagues. Some who chose not to share PIS-B talked about it being a private matter or not wanting to worry anyone. Most found PIS-B either very or somewhat useful (97/100, 97%) and either very or somewhat clear (98/100, 98%). Two participants talked about finding the concept of randomisation very difficult to understand. Some wanted more information, e.g. about extra tissue being taken, the risks and side effects of surgery, personal benefits of joining LORIS, whilst two women thought there was too much information or that it was too technical. Despite PIS-B being generally perceived as useful and clear, 39/89 (44%) of acceptors did not think it had helped them make a decision, most commonly because they had already decided to join the trial.

Most of those interviewed (70/100, 70%) had watched the patient information film and often shared it with a family member, friend or colleague (45/70, 64%). Many who did not watch the film had already made their decision or did not have a DVD player and/or access to the internet. All film viewers found it either very or somewhat clear and useful. The part of the film which was most commonly cited as being particularly useful (37/70, 53%) was the Q&A section. Very few, 4/70 (6%), identified aspects of the film as being unhelpful. Two were concerned about terminology, one woman noted she already knew some of the information from the PISs and one felt some of the questions in the Q&A session were poor. Despite positive opinions from the film viewers, there was a 50:50 split in terms of whether the film helped them decide whether to join the trial; most who did not feel the film had helped with decision making had already decided what to do before viewing it. Nevertheless, many described feeling reassured that they had made the right decision having watched the film, e.g. “I’d already decided to take part at that point. DVD was really important for me to see the doctors’ faces, who are doing the study, Good that the DVD is very short, but really helpful. Made me feel part of something.” (P88)

Three additional questions were included in the final 65 interviews, if relevant. One asked about the timing of the PIL: one woman felt it had been given too early but all the other women who remembered receiving it felt it was about right. Some talked about finding the PIL useful because they had been shocked after receiving their mammogram results but appreciated having written information to take away and digest. The other two questions related to whether the women were aware that their biopsy results could make them ineligible for LORIS and how clear they felt this information was in the PIL. Most of the women who answered these questions knew that their eligibility for LORIS depended on their biopsy results (56/61, 92%) and felt that information explaining this in the PIL was either very or somewhat clear (47/51, 92%). Some highlighted how helpful it had been for an HCP to also explain everything orally.

### Sites

Initially, four communication workshops were held, two in London, one in Birmingham and one in Wycombe, with a total of twenty delegates attending. Two further workshops, both in London, held as part of the Recruitment Recovery Plan, were attended by 17 health care professionals. The delegates were a mixture of trial coordinators, research nurses, radiologists, radiographers, and surgeons. Analysis of the pre- and post- workshop questionnaires resulted in a significant improvement in knowledge (*t* = 4.7, d.f. = 28, *p* < 0.001) and confidence about discussing clinical trials in general (*t* = 3.1, d.f. = 24, *p* = 0.005) and randomisation in particular (*t* = 2.6, d.f. = 25, *p* < 0.015).

The response rate to the postal survey about which aspects of the LORIS protocol was perceived as challenging was good from PIs, 34/47 (72%), and site leads, 45/53 (85%), but poor from other members of the recruiting team, 12/197 (6%). Most respondents reported they were well aware of the aims of LORIS (88/91, 97%) and comfortable with these (87/91, 96%). However, only about half stated they themselves would participate in LORIS, if relevant, or encourage a friend or relative, (45/87, 52%), many were unsure (35/87, 40%) and a few would not participate (7/87, 8%).

Most respondents were clear about the study logistics pathway (78/91, 86%) and thought the process of obtaining histopathology slides for central review was streamlined (64/85, 75%). However, the need for a second biopsy to confirm eligibility was sometimes or often thought to deter team members from recruiting patients by nearly half of respondents (37/83, 45%), and sometimes or often thought to act as a deterrent to potential participants by most respondents (68/83, 82%). Another challenge for sites was changes in their key staff since LORIS opened at their site, reported by 38/89 (43%) of respondents.

When asked about the most challenging aspect of recruiting patients (free text item), the most common reason given was the low number of eligible patients (34/81, 42%). Additionally, some respondents (12/81, 15%) noted that many patients were deemed ineligible following central review. The second most common barrier, reported by 18/81 (22%), was that many women had a strong preference for a particular treatment arm. The challenge of trying to explain active surveillance (7/81, 9%), and local logistic issues (6/81, 7%) were also barriers reported by more than one respondent. In the semi-structured phone interviews, held with 20/47 PIs (43%) and 40/53 site leads (75%), the same barriers were identified more frequently. These interviews also provided an opportunity for participants to describe in more detail specific local challenges. Some interviewees suggested that it would be beneficial to educate the general public about DCIS and the LORIS trial in general. There was also an issue noted with the use of the word carcinoma in DCIS and the use of the term ‘cancer’ in the patient information provided by hospitals, in that this could lead to more women having a strong preference for surgery.

Based on the information gained from the survey and interview, refresher SIVs were suggested for six sites and attendance at a communication workshop for 14 sites. Online refresher SIVs were held for all six sites but only five sites sent delegates to a communication workshop.

## Discussion

From the outset of the LORIS study, it was anticipated that recruitment might be difficult because the two arms (conventional treatment and active monitoring with annual mammography) could be perceived as comparing ‘something’ with ‘nothing’ [12] and much existing patient information, provided by cancer charities and hospitals, described low-grade DCIS in a manner that implied that the pre-cancerous condition would inevitably develop into invasive cancer [18]. Despite employing several best practice approaches, initial recruitment targets were not met. The data presented in this paper provide some evidence about why this was the case and help inform recommendations for future similar trials.

Since the LORIS trial opened to recruitment, a Cochrane review highlighting factors that impact recruitment to health care RCTs has been published [24]. The review lists 22 findings and many of these mirror the LORIS experience, including the decision around participation being discussed with a range of other people, the difficulty of the concept of randomisation, the potential benefits trial participation may bring and altruism as an important influencing factor. Communication of trial information was identified as a subtheme in the Cochrane review. The importance of trial information being robust, concise and jargon free, delivered verbally by a skilled, knowledgeable, person-centred individual, with written information provided as an adjunct to this, were all review findings, along with consideration about the timing of trial information. These good practice strategies were employed in the LORIS trial. Written trial information was provided in stages, rather than all at the start to prevent patients being overwhelmed. As well as the written information, potential participants also had the opportunity to watch a film about and could discuss the trial with a trained staff member. All trial materials were informed by patient and public focus groups to ensure they were clear, concise and jargon free.

The Cochrane review also emphasised the importance of tailoring the invitation to participate in a trial to each individual. This was reflected in the findings for LORIS: some women described reading everything very carefully and doing additional research, whilst others skimmed through the written materials; some wanted more detailed information whilst some wanted shorter, less technical information. Strategies for providing trial information in a more flexible way should be considered in the future to try to meet the needs of potential participants who have different preferences and different levels of health literacy. As well as providing information in different modalities, the provision of information with different levels of detail should be considered and this could be facilitated by digital information provision. A recent systematic review of digital tools in the informed consent process found that whilst there was no evidence of negative outcomes, more research was required to confirm benefit [25]. There was some evidence in this review to suggest that multimedia tools might result in better outcomes, compared with videos, perhaps because they are more interactive.

As well as allowing individuals to tailor information provision, digital tools also have the potential to reduce the risk of researcher bias, i.e. the researcher influencing the patient’s decision on participation. This is important for a study like LORIS: about half of site staff reported that, if they were eligible for LORIS, they would either not take part or were unsure. Although the assumption of clinical equipoise underpins RCTs, these findings indicate that many site staff had a preconceived preference for a treatment arm, and this may have impacted recruitment. Although digital tools may benefit the informed consent process, it is important to consider the needs of potential participants with a low level of digital literacy and ensure they have sufficient support to use these tools.

Whilst the LORIS patient information materials were well received, many women did not think these had influenced their decision about whether to accept randomisation. Nor did most women feel that their decision had been influenced by discussions with HCPs. Of those who were registered in LORIS and remained eligible following central review, 81% agreed to be randomised, even though randomisation was a concern for nearly half of those women. The major drivers for participation were the belief that the trial offered the best treatment and altruism. The women who withdrew after randomisation did so because they did not want surgery and had been hoping to be randomised to the active monitoring arm. Most of the women who declined randomisation were clear that they wanted surgery, either because of their own concerns or concerns of others. Clear individual patient preference for how DCIS is managed was the reason that the trial design for the LORD study was amended from an RCT to a patient preference design [26]. Anxiety levels may influence patient preference. The baseline measures in LORIS suggest that whilst participants were reasonably healthy both on physical and mental health scores, a relatively high proportion of women had high levels of state anxiety (43%) and nearly a third had high levels of trait anxiety. It will be important to compare and explore anxiety levels longitudinally in the two arms of LORIS to test whether there are any differences.

Communication workshops for site staff improved knowledge about LORIS and confidence to discuss the trial, but it was not possible to explore whether this improved recruitment rates, given other variables, e.g. staffing levels, which affect recruitment. Site staff suggested that low recruitment was due to the stringent inclusion criteria, meaning there were low numbers of eligible patients, and where applicable, the need for a second biopsy, as well as women’s own preferences. Some staff suggested that providing education to the public about DCIS and presenting it outside the cancer context could influence patient decision making.

## Limitations of the study

Some women declined to take part in the study when first approached. Data were not collected from these individuals and it may be that their reasons for declining trial entry were different from those women who declined randomisation. The number of women who declined participation when first approached about the study will be reported in the main trial results paper. Less than half of those who declined to be randomised completed a CTQ so some caution is required in generalising from the CTQ and interview results presented here on decliners. Education level was not collected so it is not possible to explore whether this affected the decision to participate in the trial.

## Conclusions

Recruitment to LORIS was challenging despite employing recommended strategies aimed at patients and site staff which were well received by both groups, and improved staff knowledge and confidence. A more flexible approach to information provision, tailoring to individual needs and preference, would support informed decision making and may improve future recruitment. Crucially, before recruitment opens, all trial staff need to be fully on board with the trial. How can we expect trial staff to recruit others if they would not participate themselves?

### Supplementary Information

**Additional file 1.** Patient interview schedule.

**Additional file 2.** Chapters in the LORIS Patient Information Film.

**Additional file 3.** LORIS Communication Workshop Protocol.

**Additional file 4.** Questionnaire completed by staff before the workshop.

## Data Availability

The datasets used and analysed during the current study are available from the corresponding author on reasonable request.

## Abbreviations

COMET: Comparison of Operative to Monitoring and Endocrine Therapy Trial
CTQ: Clinical trials questionnaire
DCIS: Ductal carcinoma in situ
EQ-5D: EuroQOL 5D-5 L
HCP: Health care professional
LORD: Low Risk DCIS Study
LORETTA: Low-risk DCIS with endocrine therapy alone-tamoxifen
LORIS: Low-risk DCIS
MCS: Mental component summary
NHS R&D: National Health Service Research and Development
PCS: Physical component summary
PIL: Patient information leaflet
PIS: Patient information sheet
Q&A: Questions and answers
RCT: Randomised controlled trial
SF-36v2: Short Form Health Survey- 36 version 2
SIV: Site initiation visit
STAI: Spielberger State Trait Anxiety Inventory
VACB: Vacuum-assisted core biopsy
