# Supplementary material for: Recruiting women with ductal carcinoma in situ to a randomised controlled trial: lessons from the LORIS study
Source: Trials. 2023 Oct 14;24:670. doi: 10.1186/s13063-023-07703-4 (PMC10576350; doi:10.1186/s13063-023-07703-4)
Supplement: Supplementary file 1 — Additional file 1. Patient interview schedule. [file 13063_2023_7703_MOESM1_ESM.docx]

**Supplementary Material 1: Patient interview schedule**

Introduce yourself as a researcher from SHORE-C and the reasons why you are phoning. Explain that the interview will last approximately 15 minutes and that we are interested in their decision regarding trial participation. Check it is convenient to continue and remind them the interview is confidential.

**Section 1: About you**

First I’d like to ask a few general questions about you

1. **Date of Birth:** ____ / ____ / ____
2. **Marital status:**

Single ❑ Married/Partner ❑ Divorced/Separated ❑ Widowed ❑

1. **Employment Status:**

Unemployed ❑ Retired ❑ PT ❑ FT ❑ Self-employed ❑

1. **Can I ask if you are taking part in the LORIS Study?**

Taking part ❑ Not taking part ❑

1. **Who discussed the trial with you?** (tick all that apply)

Research Nurse ❑ Radiologist ❑ Oncologist ❑ Surgeon ❑

Other ❑

If other please state

1. **Did any of these health care professionals influence your decision to take part or not? Let patient respond freely**

Research Nurse Yes ❑ No ❑

(If yes can you tell me in which ways)

Radiologist Yes ❑ No ❑

(If yes can you tell me in which ways)

Oncologist Yes ❑ No ❑

(If yes can you tell me in which ways)

Surgeon Yes ❑ No ❑

(If yes can you tell me in which ways)

Other Yes ❑ No ❑

If other please state

(If yes can you tell me in which ways)

**Section 2a: Information received (PIS)**

Now I would like to ask about the trial information you received and what you thought about it.

1. **Did you receive any of the following LORIS Study Patient Information Sheet (s)?** Remind patient when they would have received each sheet.

Following mammogram (PIL) ❑ Registration (A) ❑

Randomisation (B) ❑

I don’t know/I don’t remember ❑(Skip to Section 2b Q16 if this response given)

1. **Did you read any of them?**

Yes ❑ No ❑(ask reasons why & ask Q10)

1. **If yes which ones**

Following mammogram (PIL) ❑ Registration (A) ❑

Randomisation (B) ❑

I don’t know ❑

1. **Thinking back to when you were given the Patient Information Leaflet, would you say it was provided:**

Too early ❑ too late ❑ about right❑

1. **Were you aware that your biopsy results may have shown you to be ineligible for LORIS?**

Yes❑ No❑

**This information was contained in the Patient Information Leaflet**

1. **How clear was that piece of information in the Patient Information Leaflet?**

Very ❑ Somewhat ❑ A little ❑ Not at all ❑

*****We will now focus on this sheet (NTI randomisation/Sheet C)*****

1. **Did any of your family members, friends or colleagues read the LORIS Study patient information sheet?**

Yes ❑ No ❑

(Note whom)

1. **How useful did you find the LORIS Study Patient Information Sheet?**

Very ❑ Somewhat ❑ A little ❑ Not at all ❑

1. **How clear was it?**

Very ❑ Somewhat ❑ A little ❑ Not at all ❑

1. **Where there any parts that were not clear?**

Yes ❑ No ❑

If yes note which part (s)

1. **Did the LORIS Study patient information sheet help you make a decision about whether or not to join the trial?**

Yes ❑ No ❑ Not particularly ❑

**Section 2b: Information received (DVD)**

Now I would like to ask you a few questions about the LORIS Study patient information DVD.

1. **Were you given LORIS Study patient information DVD?**

Yes ❑ No ❑(If no ask Q16 & Q17)

1. **Were you referred to the LORIS website to watch the DVD content?**

Yes ❑ No ❑

1. **Were you not given the LORIS DVD because you don’t have:**

a DVD player ❑ a computer ❑

1. **Did you watch it?**

Yes ❑ No ❑ Parts of it ❑

(If no why not?) (List sections watched)

1. **Did any of your family members, friends or colleagues watch the LORIS Study patient information DVD?**

Yes ❑ No ❑ Parts of it ❑

(list whom) (List sections watched)

1. **How clear was the LORIS Study patient information DVD?**

Very ❑ Somewhat ❑ A little ❑ Not at all ❑

1. **How useful did you find the LORIS Study patient information DVD?**

Very ❑ Somewhat ❑ A little ❑ Not at all ❑

1. **Which parts of the DVD did you find particularly useful?**

List parts

1. **Were any aspects of the LORIS Study information DVD unhelpful?**

Yes ❑ No ❑ Parts of it ❑

(Please list parts)

1. **Did the LORIS Study information DVD help you make a decision about whether to join the trial or not?**

Yes ❑ No ❑ Not particularly ❑

1. **Which aspect of the LORIS Study information DVD did you find most useful?**

**Section 3: Accept/Decline**

Have the participants Accept/Decline questionnaire to hand, note their response to the most important reason and ask them to explain more about this reason.

Now I’d like to ask you a few questions about how you made a decision about participating in the LORIS Study.

I noticed you said that _____ was the most important reason why you accepted/declined the LORIS Study. Please can you tell me a bit more about this?

**Final question, was there anything else you would have liked to have known before joining the LORIS Study?**

**Thank you so much for taking time to talk with me today**
