# Supplementary material for: Recruiting women with ductal carcinoma in situ to a randomised controlled trial: lessons from the LORIS study
Source: Trials. 2023 Oct 14;24:670. doi: 10.1186/s13063-023-07703-4 (PMC10576350; doi:10.1186/s13063-023-07703-4)
Supplement: Supplementary file 2 — Additional file 2. Chapters in the LORIS Patient Information Film. [file 13063_2023_7703_MOESM2_ESM.docx]

**Supplementary Material 2: Chapters in the LORIS Patient Information Film**

| **Introduction**  Introduces LORIS and explains the purpose of the DVD |
| --- |
| **What is DCIS?**  Describes DCIS using identical language to the PIS |
| **Why the LORIS Trial is so important**  Contains an interview with the LORIS Chief Investigator (CI) explaining the back ground of DCIS, overtreatment and how research in the form of a clinical trial is necessary |
| **Description of the study**  A comprehensive and step by step explanation of study the procedures |
| **Randomisation**  Uses simple graphics and patient friendly language that is complementary to the PIS to describe the process of randomisation |
| **The Groups**  Comprises information about the processes involved in each arm of the trial, standard treatment or active monitoring |
| **What else can you expect if you join the study**  Explains the importance of completion of the quality of life questionnaires |
| **Questions and answers**  A panel of simulated patients asking the LORIS CI questions about the joining LORIS |
| **Grades of DCIS**  A doctor explains the different grades of DCIS and that high grade DCIS is treated with surgery |
