# Supplementary material for: Recruiting women with ductal carcinoma in situ to a randomised controlled trial: lessons from the LORIS study
Source: Trials. 2023 Oct 14;24:670. doi: 10.1186/s13063-023-07703-4 (PMC10576350; doi:10.1186/s13063-023-07703-4)
Supplement: Supplementary file 3 — Additional file 3. LORIS Communication Workshop Protocol. [file 13063_2023_7703_MOESM3_ESM.docx]

**Supplementary Material 3: LORIS Communication Workshop Protocol**

**Aim**: To improve knowledge about LORIS recruitment and to improve communication with patients being invited to participate, including ensuring the consistency of information.

**Audience:** Healthcare professionals, including surgeons, oncologists, radiologists, research nurses actively engaged in LORIS trial recruitment. Must be familiar with patient information sheets and patient information DVD prior to attendance.

**Pre-course:** Delegates complete a pre-course questionnaire asking about their knowledge and how confident they are discussing the LORIS Study with potential patients. Questionnaire includes detailed questions about what the individual understands about the trial; what their involvement is in the trial; communication with patients; confidence around talking to patients about the trial, what team members are involved.

**Workshop content**

**1. Difficulties and challenges recruiting to breast cancer trials (group exercise)**: A short interactive group exercise to relax the group and encourage them to engage in the workshop whatever their role. Each delegate is asked to list the type of patient they find most difficult to approach about trials e.g. angry patients, ‘internet’ patients, focusing on the LORIS Study.

**2. Enhancing Informed and educated consent for LORIS (interactive presentation)** including:

1. Impact of breast screening and what is DCIS
   1. Increase in incidence of DCIS since the introduction of breast screening
   2. The Marmot report
   3. DCIS and possibility of overtreatment
2. Pre-LORIS focus group findings
   1. Views of 22 healthy women of breast screening age about knowledge of DCIS
   2. Views about joining a trial like LORIS
3. How best to describe DCIS
   1. How do the breast team describe DCIS
   2. Breast cancer charities and breast screening service description of DCIS
   3. LORIS DCIS descriptor based on published research
4. Drivers and barriers to LORIS participation
   1. Common barriers and drivers to RCTs
   2. Barriers and potential drivers to LORIS participation
   3. How to overcome these barriers
5. Handling uncertainty
   1. How to handle uncertain situations in the context of clinical trials
   2. The concept of clinical equipoise
6. Discussing randomisation
   1. Research about the difficult concept of randomisation
   2. Personality types and attitudes of patients towards randomisation
   3. How best to describe randomisation

# 3. Interactive sessions

# i) Handling difficult questions: The LORIS trial DVD & patient information materials are used. Discussion about DVD content, when and how to share DVDs with patients. The importance of communication and consistency, key points to cover.

# ii) Simulated patient exercise: Simulated patients (actors) ask delegates typical questions that potential LORIS participants may bring. Individuals formulate responses with help from the group and facilitators.

# iii) Communication challenges with patients and relatives: The facilitator uses group exercises to help identify difficulties and strengths in communicating about the LORIS trial. From these exercises the group provide feedback and discuss any recruitment difficulties to LORIS and construct consistent responses as a group.

**4. Post-Course:** Key points are summarised. Delegates complete post course questionnaires to validate whether main learning points have been achieved.
