# Supplementary material for: Recruiting women with ductal carcinoma in situ to a randomised controlled trial: lessons from the LORIS study
Source: Trials. 2023 Oct 14;24:670. doi: 10.1186/s13063-023-07703-4 (PMC10576350; doi:10.1186/s13063-023-07703-4)
Supplement: Supplementary file 4 — Additional file 4. Questionnaire completed by staff before the workshop. [file 13063_2023_7703_MOESM4_ESM.docx]

**Supplementary Material 4: Questionnaire completed by staff before the workshop**

**CONFIDENTIAL**

**Breast Cancer Surgical Trials –Pre Workshop Questionnaire**

Name: Date:

**Please indicate [ X ] your response to each question:**

1. How aware are you about your team’s trial portfolio?

**Very Somewhat Not Very**

**[ ] [ ] [ ]**

1. How aware are you about the primary aims of the **LORIS t**rial?

**Very Somewhat Not Very**

**[ ] [ ] [ ]**

1. How clear are you about the trial logistics for the **LORIS** trial as they affect your team?

**Very Somewhat Not Very**

**[ ] [ ] [ ]**

1. How clear are you as to how patients are identified as eligible for this trial?

**Very Somewhat Not Very**

**[ ] [ ] [ ]**

1. How aware are you as to who first discusses possible trial entry with patients?

**Very Somewhat Not Very**

**[ ] [ ] [ ]**

1. How aware are you about the capacity of the histopathology/ radiology department to run tests at the timepoints required for this trial?

**Very Somewhat Not Very**

**[ ] [ ] [ ]**

1. How aware are you about the screening tests that may be required to establish eligibility for this trial?

**Very Somewhat Not Very**

**[ ] [ ] [ ]**

1. How aware are you as to who obtains informed consent from the patients?

**Very Somewhat Not Very**

**[ ] [ ] [ ]**

1. How aware are you as to who informs patients which arms of the trial patients have been randomised to receive?

**Very Somewhat Not Very**

**[ ] [ ] [ ]**

1. How aware are you about the number of follow up tests required for the **LORIS** trial?

**Very Somewhat Not Very**

**[ ] [ ] [ ]**

1. How aware are you of the follow up time period of the trial?

**Very Somewhat Not Very**

**[ ] [ ] [ ]**

1. How aware are you as to who the patients can contact with questions about the trial?

**Very Somewhat Not Very**

**[ ] [ ] [ ]**

1. How aware are you about the content of the patient information sheets for this trial?

**Very Somewhat Not Very**

**[ ] [ ] [ ]**

1. How aware are you as to when the patient information sheets are given out?

**Very Somewhat Not Very**

**[ ] [ ] [ ]**

1. How aware are you as to who gives the information sheets to the patients?

**Very Somewhat Not Very**

**[ ] [ ] [ ]**

1. How aware are you of your colleagues’ informational roles concerning clinical trials?

|  | **Very aware** | **Somewhat**  **aware** | **Not aware** |
| --- | --- | --- | --- |
| Surgeon |  |  |  |
| Oncologist |  |  |  |
| Histopathologist |  |  |  |
| Radiologist |  |  |  |
| Clinical Nurse Specialist |  |  |  |
| Research Nurse |  |  |  |
| Other (please specify) |  |  |  |

1. How many patients do you think your team has recruited to trials during the past 6 months?

**No idea 0 1-5 5-10 >10**

**[ ] [ ] [ ] [ ] [ ]**

1. Have you watched the LORIS DVD?

**Yes [ ] No [ ]**

**Physical Copy [ ] On YouTube [ ]**

**Part 2:**

**ONLY For those who may have a discussion with patients about trials**

1. **How confident are you about discussing clinical trials in general with patients with cancer**?

Very Not at all

10 9 8 7 6 5 4 3 2 1

1. **How easy do you find describing randomisation?**

Very Not at all

10 9 8 7 6 5 4 3 2 1

1. **Please list the types of trials you find most difficult to discuss.**
2. **Please list the types of patients with whom you find it difficult to discuss trials.**
